# Immunoglobulin abnormalities are frequent in patients with lupus nephritis

**DOI:** 10.1186/s41927-019-0079-2

**Published:** 2019-08-21

**Authors:** M. J. Cuadrado, I. Calatayud, M. Urquizu-Padilla, S. Wijetilleka, S. Kiani-Alikhan, M. Y. Karim

**Affiliations:** 1 Louise Coote Lupus Unit, Guy’s & St Thomas’ Hospitals, London, UK; 2 Dept of Immunology, University Hospital Wales, Cardiff, UK; 3 Dept of Immunology, Barts Health NHS Trust, London, UK; 4 Dept of Pathology, Sidra Medicine, Doha, Qatar

**Keywords:** Immunoglobulins, SLE, Lupus nephritis, Hypogammaglobulinemia, Rituximab, Autoimmune, B-cells

## Abstract

**Background:**

Hypogammaglobulinemia is a complication of B-cell targeting therapies (BCTT), used in vasculitis, rheumatoid arthritis and systemic lupus erythematosus (SLE). Since autoimmune diseases are associated with underlying and induced immune abnormalities, several societies recommend assessing immune function before and during rituximab treatment. In SLE, polyclonal hypergammaglobulinemia is the typical alteration of gammaglobulins, though hypogammaglobulinemia has also been reported.

**Methods:**

This is a cross-sectional study describing immunoglobulin levels measured as part of routine care in patients with lupus nephritis, a group with multiple factors contributing to immunoglobulin abnormalities, including immune dysregulation, immunosuppression and nephrotic syndrome.

**Results:**

Polyclonal hypergammaglobulinemia occurred in 15/83 (18.1%) patients. In contrast, low levels of immunoglobulins were found as follows: selective IgA deficiency 2/83 (2.4%), reduced IgG levels 7/83 (8.4%), reduced IgM 14/83 (16.9%). Only 1 patient required immunoglobulin replacement.

**Conclusions:**

Immunoglobulin abnormalities are frequently found in lupus nephritis, ranging from polyclonal hypergammaglobulinemia to hypogammglobulinemia. Consequently, immunoglobulin levels should be assessed prior to commencing BCTT.

## Background

With widespread use of B-cell targeting therapies (BCTT), hypogammaglobulinemia is gaining recognition as a potential complication [1, 2]. BCTT is used in a range of autoimmune rheumatic diseases (AIRD), including anti-neutrophil cytoplasmic antibody (ANCA)-associated vasculitis (AAV), rheumatoid arthritis (RA) and systemic lupus erythematosus (SLE). The American Academy of Asthma, Allergy, and Immunology emphasized checking baseline immune function before rituximab treatment in autoimmune disease [3]. The European League against Rheumatism guidelines in AAV and RA recommend testing serum immunoglobulins before each rituximab course and in those developing recurrent infection [4, 5]. The British Society for Rheumatology guidelines for rituximab in RA recommend testing immunoglobulin levels before commencing treatment, 4–6 months after infusions and before re-treatment [6]. However, there is a lack of data regarding the nature of immunoglobulin abnormalities which might be found at baseline prior to starting BCTT.

Various immunoglobulin abnormalities are reported in SLE. Polyclonal hypergammaglobulinemia is well described. Hypogammaglobulinemia has been associated with SLE itself, with immunosuppression, and nephrotic syndrome [7]. Both IgA and IgM deficiencies have been reported in SLE [8]. Therefore, it is important when monitoring immunoglobulins as recommended above, to be aware of the likely baseline immunoglobulin results. In this report, we describe results of a cross-sectional study of immunoglobulin levels in lupus nephritis. This group is likely to have multiple factors contributing to immunoglobulin abnormalities (including corticosteroids, immunosuppressive agents, severe nephrotic syndrome, and associated immunodysregulatory disorders) [1, 7–9]. These patients will usually require immunosuppressive therapy, which may include BCTT. Here, in this concise communication, we report immunoglobulin levels in our lupus nephritis cohort to illustrate the range of abnormalities which might be found prior to commencement of BCTT.

## Methods

Patients with SLE, and lupus nephritis were included in this short report. All patients fulfilled at least 4 ACR classification criteria for SLE [10], and attended the Louise Coote Lupus Unit at Guy’s and St Thomas’ Hospitals, London, United Kingdom between 2009 and 11. The diagnosis of lupus nephritis was established by renal biopsy with light, electron, and immunohistochemical microscopy, and reported according to the 2004 International Society of Nephrology/Renal Pathology Society classification criteria. Patients received a range of immunosuppressive medications including hydroxychloroquine, corticosteroids, azathioprine, mycophenolate mofetil. Those patients who had received cyclophosphamide, were treated with the EuroLupus lower dose protocol. They had not received multiple courses of BCTT, which could have specifically affected memory B-cell numbers, and consequently immunoglobulin results.

We routinely measured immunoglobulins (IgG, IgA, IgM) and serum protein electrophoresis in adult patients with biopsy-proven lupus nephritis. Immunoglobulins were measured by an immunoturbidimetric assay on the Roche Modular Analytics system (Roche Diagnostics GmbH, Mannheim, Germany), and electrophoresis was performed on agarose gel. The results were considered as follows: for IgG and IgM isotypes, low results were below the lower limit of the normal reference range for the assay (as established by the Guy’s & St Thomas’ Chemical Pathology Department). For IgA, a level of < 0.07 g/L was considered in keeping with the international definition for selective IgA deficiency [11]. Ethical approval was not necessary for immunoglobulin testing at our institution as all such tests were requested as part of routine care of the patients. The patients were enrolled in a study of serological markers of disease activity in SLE, ethical approval was granted by the South Glasgow and Clyde Research Ethics Committee, Glasgow, Scotland.

Chart review was undertaken in patients with low immunoglobulins to assess infection history and use of immunoglobulin replacement therapy (IGRT). Patients found to have low IgA or IgG levels were questioned about infection history as part of standard care, though not patients with isolated IgM deficiency, as this was not considered to be clinically significant at that time. Patients found to have low IgG levels were monitored clinically, and with repeated immunoglobulin levels in some cases.

Simple descriptive statistics are employed. There was no utilisation of complex statistical analysis in view of the small numbers of patients identified with low immunoglobulins and/or significant infection history.

## Results

### Patients

Eighty-eight patients with biopsy-proven lupus nephritis were included (3 male, 85 female). The mean age was 37.9 ± 10.9 years in this study.

### Laboratory testing

**Fig. 1:**
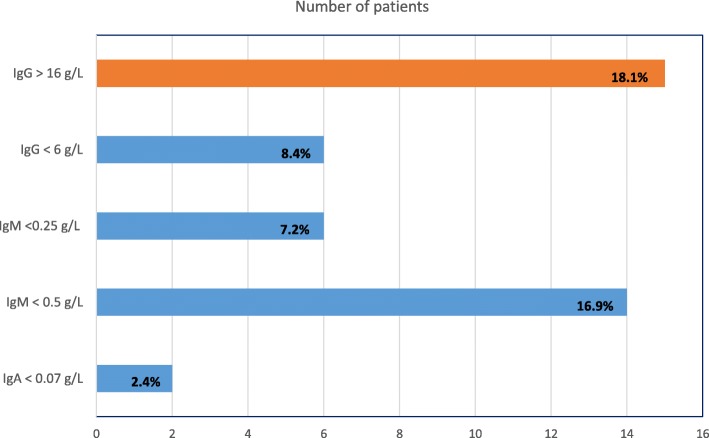
Immunoglobulin abnormalities in a group of 88 patients with lupus nephritis

Results of serum immunoglobulins were available on 83/88 patients. Polyclonal hypergammaglobulinemia with high IgG levels occurred in 15/83 (18%) patients. Conversely, low levels of immunoglobulins were found as follows: selective IgA deficiency 2/83 (2.4%), reduced IgG levels 7/83 (8.4%), reduced IgM 14/83 (17%). These results are summarized in Fig. 1. There were also overlapping patients, as 4 patients with IgM deficiency also had reduced IgG levels. Because this report reflects tests routinely ordered in the Lupus Unit, results of specific anti-microbial antibodies are not available, as these were not usually requested by physicians.

### Clinical review

Chart review was performed on patients with low immunoglobulin results. Neither of the 2 patients with selective IgA deficiency demonstrated a history of recurrent infections. Only 1/7 patients with low IgG had a significant recent infection history, but had also undergone previous splenectomy. This patient was reviewed by Clinical Immunology, and IGRT was indicated after clinical assessment. IGRT by the intravenous route was commenced at 0.4 g/kg/month. Monitoring of the low IgG group revealed that in another two of these patients, IgG levels increased towards normal, in one case with improvement of nephrotic syndrome, and in another patient over time (with reduction of corticosteroid dose and passage of time since cyclophosphamide).

## Discussion

Immunoglobulin abnormalities are frequent and diverse in patients with lupus nephritis. The most frequent finding is of elevated immunoglobulin levels, consistent with the generalised increased immune activity found in SLE. Such a pattern is also well recognised in Sjogren’s syndrome. Although non-specific, the finding of polyclonal hypergammaglobulinemia can be a further laboratory clue for connective tissue disease in undiagnosed patients.

Neither selective IgA nor IgM deficiency are usually associated with recurrent infections, and do not typically require intervention with IGRT. Selective IgA deficiency is defined as IgA < 0.07 g/L, with normal IgG and IgM levels [11]. This is different to partial IgA deficiency where the IgA level is below the lower limit of the reference range, but still detectable. In selective IgA deficiency, it is important to be aware of the very small risk of reaction to transfusions and immunoglobulin infusions (due to contamination with small amounts of IgA which these patients recognize as foreign). Patients with partial IgA deficiency do not have this risk, as they are tolerant to IgA. Selective IgA deficiency is known to be associated with SLE: Cassidy et al. reported prevalence of 2.6% in their cohort including 152 adults, defining IgA deficiency as < 0.01 g/L [12], while we found a 2.4% prevalence. Selective IgA deficiency is also common in the general population (1 in 223 to 1 in 1000), though the vast majority of patients with selective IgA deficiency do not have problems with infections [11].

Selective IgM deficiency has not traditionally been thought to be a major risk factor for recurrent infection, though there is more recognition recently as an immunodeficiency disorder [13]. It may be associated with more severe or long-standing SLE, and could relate to long-term corticosteroid or immunosuppressive therapy [8, 14]. We found 4/83 (4.8%) patients with both low IgM and IgG, and the risk of infection in these patients is considered to be more strongly linked to the low IgG, though some studies show that low IgM in association with low IgG can be associated with increased risk of infection-related chronic lung disease/bronchiectasis [15]. The Urowitz group in Canada showed showed consistent association between low immunoglobulin level and clinically significant infection in SLE. They reported that low IgG and IgM levels increased the risk of severe infection [16].

Although we found low IgG levels in 8.4% of our cohort, most patients in this group did not report increased infections. Common variable immunodeficiency has been associated with SLE, but is still very rare [6]. Low IgG may be reversible in some patients with immunosuppressive drug-related reduction, including non-biologic medications such as corticosteroids (17). Low IgG (though not usually IgA or IgM) can be found in severe cases of nephrotic syndrome with marked proteinuria. Smilek et al. reported hypogammaglobulinemia in 16/102 (15.7%) patients prior to initiation of induction therapy for lupus nephritis in the Abatcept and Cyclophosphamide Combination Efficacy and Safety Study (ACCESS). Serum IgG levels inversely correlated with urinary protein-creatinine ratio (7). We have reported a patient with severe SLE-related nephrotic syndrome, with IgG falling to a nadir of 1.7 g/L while urine protein excretion was 11.95 g/24 h. This improved to 4.9 g/L as the urine protein lessened to 1.19 g/24 h with treatment over a 6-month period [8].

It is most important to consider infection history, as this is the key factor in decision-making regarding immunoglobulin replacement. Monitoring of immunoglobulin levels at 6–12 monthly intervals may be appropriate. Such monitoring over time can help to distinguish between patients developing minor, transient, hypogammaglobulinemia without infections, and those less commonly, who develop a sustained reduction and clinically significant picture [2, 9, 17]. Although most hypogammaglobulinemic patients are asymptomatic, it is noteworthy that hypogammaglobulinemia has been associated with an increased infection risk in patients with AIRD in multiple studies [13]. There is data from AAV, RA, and SLE, as above [1, 16].

It is also noteworthy that low baseline IgG is a risk factor for clinically significant hypogammaglobulinemia in AIRD patients treated with BCTT [18]. During rituximab treatment, baseline IgG level at the start of maintenance therapy was the only factor associated with future development of ‘significant’ hypogammaglobulinemia, defined as IgG < 4 g/L [19]. Significant hypogammaglobulinemia only developed if baseline maintenance IgG was in the lowest quartile (4.08–5.59 g/L). Hence, the importance of measurement of immunoglobulins prior to commencing BCTT is emphasized.

Measurement of specific anti-microbial antibodies (pneumococcus, tetanus, haemophilus) is used to assess functional antibody deficiency and when appropriate, response to test vaccination is used as a diagnostic tool in patients with low specific antibody results. In patients with persistent hypogammaglobulinemia, and/or recurrent infections, and/or impaired response to test vaccination (functional antibody deficiency), early referral to Clinical Immunology is recommended [17]. We do not have data on specific antibody measurements in our cohort, as these tests were not routinely requested. When indicated, assessment of suitability for IGRT is often undertaken by Clinical Immunology, according to a recent multidisciplinary taskforce [17], due to their IGRT experience, in contrast to high dose immunomodulatory immunoglobulin [20]. The use of IGRT has been effective in management of a selected group of AIRD patients with secondary hypogammaglobulinemia [1, 2, 21]. The UK group utilized a replacement dose of 0.4 g/kg/month, the recommended dose as per the Department of Health guidelines [21, 22]. Both intravenous and subcutaneous routes are effective and safe for immunoglobulin replacement [20].

For patients with *extremely* low IgG levels (below 1–2 g/L), and evidence of functional antibody deficiency, IGRT can be considered even in the absence of recurrent infection, because of the major future risk of infection.

We acknowledge the limitations of this concise report, as we are not able to access and provide detailed individual clinical information regarding treatment schedules, disease activity, renal biopsy findings, and white cell count results. This further data would have undoubtedly provided additional useful background and context for the reader.

## Conclusions

1. Immunoglobulin abnormalities are frequent in lupus nephritis, including both elevated and low levels
2. Immunoglobulin levels should be measured, as these can be influenced by the disease, renal dysfunction, and medications
3. Immunoglobulin levels should be requested prior to commencing BCTT

## Data Availability

All important and relevant data is presented within the manuscript itself.

## Abbreviations

AIRD: Autoimmune rheumatic diseases
BCTT: B-cell targeting therapies
SLE: Systemic lupus erythematosus
